# Silencing an aphid-specific gene *SmDSR33* for aphid control through plant-mediated RNAi in wheat

**DOI:** 10.3389/fpls.2022.1100394

**Published:** 2023-01-09

**Authors:** Jiahui Zhang, Huiyuan Li, Xue Zhong, Jinfu Tian, Arnaud Segers, Lanqin Xia, Frédéric Francis

**Affiliations:** ^1^ Institute of Crop Sciences, Chinese Academy of Agricultural Sciences (CAAS), Beijing, China; ^2^ Functional and Evolutionary Entomology, Gembloux Agro-Bio Tech, University of Liege, Gembloux, Belgium; ^3^ National Nanfan Research Institute (Sanya), Chinese Academy of Agricultural Sciences/Hainan Yazhou Bay Seed Laboratory, Sanya, Hainan, China

**Keywords:** wheat (*Triticum aestivum* L), grain aphid (*Sitobion miscanthi*), RNA interference (RNAi), salivary protein, aphid control

## Abstract

Grain aphid (*Sitobion miscanthi*) is one of the most dominant and devastating insect pests in wheat, which causes substantial losses to wheat production each year. Engineering transgenic plants expressing double strand RNA (dsRNA) targeting an insect-specific gene has been demonstrated to provide an alternative environmentally friendly strategy for aphid management through plant-mediated RNA interference (RNAi). Here we identified and characterized a novel potential RNAi target gene (*SmDSR33*) which was a gene encoding a putative salivary protein. We then generated stable transgenic wheat lines expressing dsRNA for targeted silencing of *SmDSR33* in grain aphids through plant-mediated RNAi. After feeding on transgenic wheat plants expressing *SmDSR33*-dsRNA, the attenuated expression levels of *SmDSR33* in aphids were observed when compared to aphids feeding on wild-type plants. The decreased *SmDSR33* expression levels thus resulted in significantly reduced fecundity and survival, and decreased reproduction of aphids. We also observed altered aphid feeding behaviors such as longer duration of intercellular stylet pathway and shorter duration of passive ingestion in electroneurography assays. Furthermore, both the surviving aphids and their offspring exhibited decreased survival rates and fecundity, indicating that the silencing effect could be persistent and transgenerational in grain aphids. The results demonstrated that *SmDSR33* can be selected as an effective RNAi target for wheat aphid control. Silencing of an essential salivary protein gene involved in ingestion through plant-mediated RNAi could be exploited as an effective strategy for aphid control in wheat.

## Introduction

Aphids are the most destructive agricultural insect pests, which cause potential yield losses of common wheat (*Triticum aestivum* L) by sap-sucking and virus transmission (Xia et al., 2012; Sun et al., 2019). The grain aphid (*Sitobion miscanthi*) is one of the most devastating wheat aphids that causes substantial damage to wheat, which was previously misidentified as *Sitobion avenae* (Zhang et al., 2013; Yu et al., 2014; Zhang et al., 2022a). Currently, neurotoxic insecticides are still the predominant measure for aphid management. However, intensive use of pesticides can cause aphid resistance and harmfulness to non-target organisms, which leads to environmental issues (Sanahuja et al., 2011). Limited aphid resistance germplasm has significantly hampered the process of conventional breeding projects (Crespo-Herrera et al., 2019). Therefore, it is imperative to search for effective and practical strategies for aphid management in wheat.

RNA interference (RNAi) has been recognized as one of the most potential technologies for pest control. Transgenic plant-mediated RNA interference (RNAi), which provides a protective and environmentally friendly strategy for aphid management, has been proven to be a practicable method in recent years (Price and Gatehouse, 2008). For example, interference of structural sheath protein (SHP) encoding gene in grain aphids by feeding on transgenic barely plants effectively reduce their survival and reproduction rates. Knock-down of *shp* strongly affect feeding behavior and the transgenerational effect can last for the next seven generations (Abdellatef et al., 2015). The dsRNA-transgenic *Arabidopsis* plants with the cuticular protein gene impaired the fecundity of *Myzus persicae* (Bhatia and Bhattacharya, 2018). Transgenic wheat plants expressing *SaZFP*-dsRNA decreased the survival and fecundity significantly in *S. avenae* with effects also observed on offspring (Sun et al., 2019). Plastid-expressed dsRNAs can be efficiently applied for sap-sucking pest control. Aphids feeding on transplastomic plants exhibited significant mortality, decreased aphid fecundity, and reduced weight of survivors (Dong et al., 2022).

As sap-sucking insects, aphids secrete gel saliva during stylet penetration and watery saliva during sap sucking (Khan and Naveed, 2022). Aphid salivary protein plays a pivotal role in the interaction between pest and host plants (Pan et al., 2015). ApC002 was first discovered in *Acyrthosiphon pisum* and has been proven to play a critical role in the foraging and feeding process of pea aphid (Mutti et al., 2008). Transient expression of salivary proteins in *Nicotiana benthamiana*, such as Mp10, Mp42, Mp56, Mp57, and Mp58, caused reduced virulence and fecundity of green peach aphids (Bos et al., 2010; Elzinga et al., 2014; Rodriguez et al., 2014). *M. persicae* salivary proteins Mp1, Mp2, Mp55, and MpMIIF1 were verified to inhibit host plant defense responses and facilitate green peach aphid performance on host plants (Pitino and Hogenhout, 2013; Elzinga et al., 2014; Naessens et al., 2015). Overexpression of salivary proteins Me10 and Me23 enhanced potato aphid infestation and fecundity (Atamian et al., 2013). Knockdown of the transcript of effector protein Armet by RNA interference impeded the feeding behavior of pea aphids. Overexpression of *Armet* in *N. benthamiana* was shown to activate plant-pathogen interactions and induce salicylic acid-mediated defense in plants, but had no detectable effects on aphid performance (Wang et al., 2015b; Cui et al., 2019). Expression of bird cherry-oat aphid candidate effector Rp1 in transgenic barley plants significantly promoted aphid fecundity and suppressed plant defense responses (Escudero-Martinez et al., 2020). Besides, transient overexpression of salivary effectors Sm9723 and Sg2204 in tobacco inhibited cell death and suppressed plant defense responses. Silencing *Sm9723* through a nanocarrier-mediated dsRNA delivery system significantly decreased the survival rates and fecundity of aphids and affected feeding behavior. Similarly, *Sg2204*-silenced aphids exhibited a strong wheat defense response and negatively impacted aphid survival rate, fecundity, and feeding behavior. The aphid performance on host plants was significantly reduced when silencing the homologs of *Sg2204* from four other aphid species (Zhang et al., 2022a; Zhang et al., 2022b). These results implied that the genes encoding salivary proteins in aphids are potential candidates for aphid control in plants though plant-mediated RNAi.

Here, we isolated a novel putative salivary protein encoding gene, *SmDSR33*, in grain aphid based on our previous transcriptomic profiling. We found that feeding on transgenic wheat plants expressing *SmDSR33*-dsRNA decreased the survival rate and the fecundity significantly in grain aphids. The surviving aphids exhibited a silencing effect and induced a transgenerational effect on their offspring.

## Materials and methods

### Plants and insects

Plants: the hexaploid wheat variety *Triticum aestivum* L. cv Zhengmai 7698 (ZM7698) was used in this study. A total of 30-35 wild-type and transgenic wheat plant seeds were sown in pots and were cultured in a climate chamber at 22°C under a 16-h photoperiod, and with a relative humidity of 40%-60%.

Insects: grain aphids, *S. miscanthi* were reared on two-leaf stage aphid susceptible wheat seedlings in a controlled chamber with similar conditions than for plant growing. Apterous adult grain aphids from a single clonal lineage were reared on wheat seedlings in a continuous culture for 24 hours to produce synchronized nymphs. After that, the adults were removed, and the offspring were used in subsequent experiments. All experiments were carried out in a climate chamber under the above-mentioned conditions.

### Isolation and characterization of *SmDSR33*

Total RNA of pooled adults was extracted by using TransZol Up (TransGen Biotech, Beijing, China). cDNA was synthesized by using FastKing RT Kit (Tiangen, Beijing, China). The full length of the *SmDSR33* gene was obtained using TransStart^®^ FastPfu DNA Polymerase (TransGen Biotech, Beijing, China) following the instructions. The DNA amplification products were sequenced by the Institute of Crop Sciences (Institute of Crop Sciences, Chinese Academy of Agricultural Sciences, Beijing, China). The theoretical isoelectric point (pI) and molecular weight (MW) of SmDSR33 were calculated through ExPASy (https://web.expasy.org/compute_pi/). The transmembrane region and putative signal peptide were predicted using TMHMM (http://www.cbs.dtu.dk/services/TMHMM/) and SignalP (http://www.cbs.dtu.dk/services/SignalP/), respectively. The counterparts of *SmDSR33* of other aphid species were obtained by the Basic Local Alignment Search Tool (BLAST) against the aphidbase database (http://bipaa.genouest.org/is/aphidbase/). Phylogenetic trees of *SmDSR33* in twelve aphid species were constructed using the nucleotide acid sequences as a matrix *via* MEGA X software (www.megasoftware.net). The branch strength was analyzed by using the maximum likelihood method and performing 100 bootstrap replications.

### Vector construction and wheat transformation

To amplify the 439 bp *SmDSR33* target sequence, specific primers were designed. A 320 bp fragment of GFP were selected as a control in the aphid bioassay experiment. The amplified PCR products were recovered and inserted at inverted repetitions into the *Spe*I/*Eco*RV and *Sac*I/*Hpa*I sites of the pEasy-Blun-Zero-AdhI vector to construct the hairpin RNAi, Bzero-DSR33-adhI-DSR33. The vector of Bzero-DSR33-adhI-DSR33 was digested by *Ssp* I and *BsrG* I to obtain the expression cassette. The latter was recovered for bombardment. The RNAi fragment was driven by the maize Ubi promoter. Bombardment-mediated transformation was applied to immature embryos isolated from ZM7698. Somatic embryos were induced in tissue culture on medium, and whole plants were then regenerated and selected. Healthy seedlings were transplanted to soil to grow until maturity.

### Southern blot analysis

The CTAB method was used to extract genomic DNA from young T_3_ plant leaf tissues as described by Sambrook et al. (1989). The restriction enzyme was used to digest 35 μg of genomic DNA overnight. The products were fractionated for 12-16 h at 60 V on a 0.8% agarose gel in 1×TBE buffer. The Hybond-N^+^ membranes were used for blotting (Amersham, UK). The digoxigenin (DIG) High Prime DNA Labeling and Detection Starter Kit I (Roche, Germany) was used for prehybridization, hybridization, washing, and detection of the membranes. The primer sets SmDSR33S-F/R were used to synthesized DNA probes (**Supplementary Table 1**).

### Quantitative real-time PCR

For the expression level of *SmDSR33* at different development stages, total RNAs of grain aphids were isolated from the four nymphs and adults reared on susceptible wheat. For the expression level of *SmDSR33* in aphids fed with different transgenic wheat and wild-type plants, the adult aphids were collected and used for total RNA extraction and further experiments.

The cDNA was synthesized following conventional procedures. A quantitative real-time PCR (qRT-PCR) assay was carried out using the SYBRH Green Real-time PCR Master Mix (Tiangen, Beijing, China) in an ABI 7300 Real Time PCR system. The aphid *Actin* gene and ribosomal protein S27 A (*Rps27*) gene were selected as internal controls, and *SmDSR33* specific primers were designed for normalization (**Supplementary Table 1**). All qRT-PCR experiments were performed in triplicate. The relative gene expression of each target gene was calculated by using the mean value of the reference genes through the 2^–ΔΔCT^ method (Livak and Schmittgen, 2001).

### Aphid bioassays

A single clonal lineage of apterous adult grain aphids was reared on wheat seedlings in cages for 24 hours to produce nymphs. The newborn nymphs produced during the period of 24 hours were transferred to fresh transgenic wheat plants.

T_3_ homozygous wheat plants with *SmDSR33*-dsRNA expression were selected to evaluate the effects on aphid survival and fecundity. At the 3-4 leaf stage, 20 neonatal first instar nymphs of *S. miscanthi* were placed on the leaf of each plant. The mortality of aphids was recorded every day. Ten plants from each line were used in every experiment. The experiment was repeated three times.

Life cycle parameters were calculated as follows: the net reproductive rate, *R*_0_= ∑^​^ *l*_*x*_·m_*x*_ , the mean generation time, *T*=∑^​^ *xl*_*x*_ *m*_*x*_/∑^​^ *L*_*x*_ *m*_*x*_ the intrinsic rate of increase, *r*_*m*_=(*lnR*_0_)/*T* , and the finite rate of increase, *λ* =*e* ^*r*_*m*_^ . In the equations, *l*_*x*_ is the surviving rate to a specific age *x* , and *m*_*x*_ is the number of new-born nymphs produced by per live adult for a specific age *x* (Biondi et al., 2013).

### Electrical penetration graph technique analysis

The Giga-8 DC EPG amplifier (EPG-Systems, Wageningen, Netherlands) and a Faraday cage was used to record the probing and feeding behaviors of apterous adult aphids on wheat. Firstly, synchronous adult aphids were inoculated on 33-592 transgenic plants and control plants for two days, respectively. Then, the aphid was starved for 2 h. After that, water-soluble silver conductive paint was used to attach each aphid to a flexible gold wire (18 μm diameter×2 cm length) through the dorsal thorax individually. The aphids were placed onto the adaxial side of a leaf from transgenic and wild-type wheat plants at the three-leaf stage, and the opposite ends of the gold wires (2 mm in diameter×3 cm length) were connected to copper wire with conducting silver glue, which was connected to a DC amplifier. The plant electrode was inserted into the soil. Under light conditions, the EPG signal of each individual was continuously monitored for 8 h. We monitored 12 behavioral recordings for each treatment. The software Stylet+a (EPG-Systems) was used to analyze EPG signals. According to the method described by Tjallingii (Tjallingii, 1985; Tjallingii, 1994), the different waveforms were correlated with feeding behavior. Non-probing (np) waveform, which reflects stylet external to wheat leaf tissue. Pathway phase contains two waveforms, waveform C, which reflects the intercellular stylet pathway, potential drops (pd), which reflects intracellular punctures during intercellular pathway. Waveform G (xylem phase) is the only waveform that reflects active sap ingestion from xylem elements. Phloem phase can be divided into two phases: E1 always occurs at the start of the phloem phase and reflects saliva secretion into the sieve element, E2 reflects passive phloem sap ingestion. EPG data was analyzed using the EPG-Excel data workbook provided by Sarria et al. (2009).

### Statistical analysis

The two-tailed Student’s *t*-test was used to evaluate the differences between wild-type and transgenic wheat lines. For all comparisons, significance (*P* value) was calculated at the 1% or 5% level. The standard error of the mean (SEM) for each treatment was calculated using three biological replicates. For the EPG experiments, means and standard errors of variables were calculated from recordings per individual aphid, and differences were analyzed by Student’s *t*-test. All data represents means ± SEM.

## Results

### Characterization of *SmDSR33* gene in grain aphids

**Figure 1:**
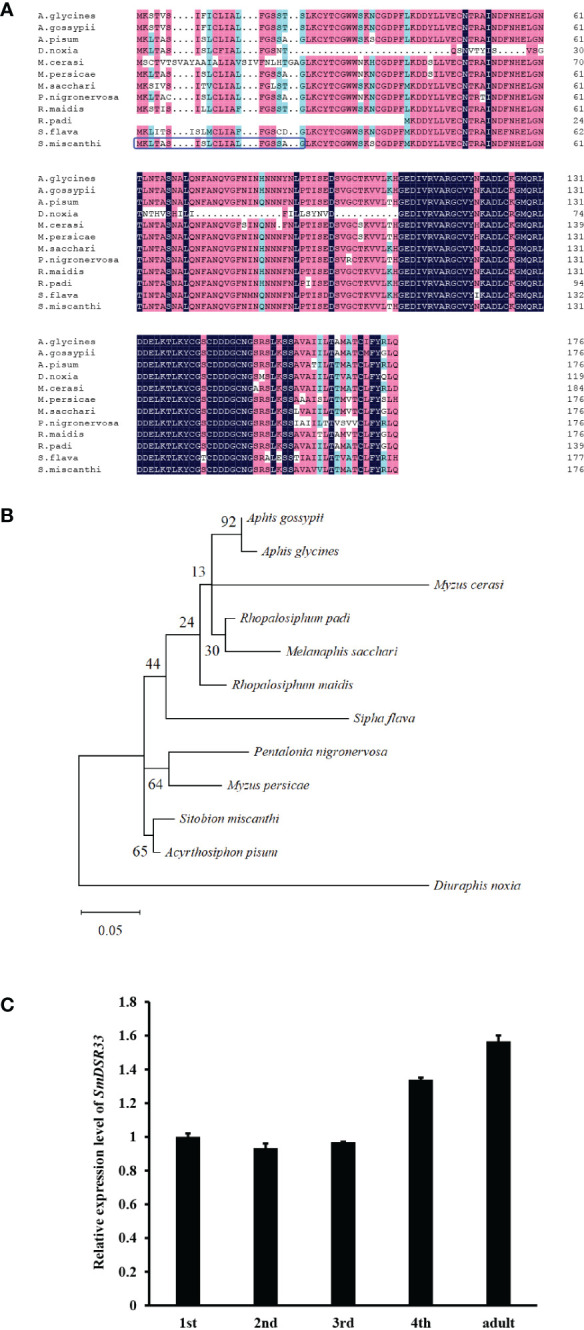
Characterization of SmDSR33. **(A)** Multiple sequence alignment of SmDSR33 protein and orthologs from other aphid species. The deduced amino acid sequences from eleven aphid species include *Acyrthosiphon pisum* (NM_001163178.1), *Aphis glycines* (AG000929-RA), *Aphis gossypii* (XM_027996540.1), *Diuraphis noxia* (XM_015509488.1), *Melanaphis sacchari* (XM_025349621.1), *Myzus cerasi* (Mca00769.t1), *Myzus persicae* (XM_022311485.1), *Pentalonia nigronervosa* (g3912.t1), *Rhopalosiphum maidis* (XM_026949227.1). *Rhopalosiphum padi* (Rpa07522.t1), and *Sipha flava* (XM_025560312.1). Black shades indicate identical amino acids. Pink shades indicate similar amino acid, and blue shades include the sequences with identical and similar residues. Signal peptide of SmDSR33 is highlighted with blue box. **(B)** Phylogenetic tree of SmDSR33 and its homologs from other aphid species constructed with the maximum likelihood method. Bootstrap supporting values (1000 replicates) are shown at the branch nodes. **(C)** The expression profile of *SmDSR33* in grain aphid at different development stages. The expression profiles of *SmDSR33* at different developmental stages of grain aphids fed on wheat. Values and error bars represent the mean and SEM of three independent biological replicates, each with a pool of 15 individual aphids.

We identified a candidate gene *SmDSR33*, which encoding a putative salivary protein in grain aphid, based on transcriptomic profiling and dsRNA feeding assay (Wang et al., 2015a). The full-length cDNA sequence of *SmDSR33* was 534 bp in length, encoding a 177 amino acid putative salivary protein. The SmDSR33 protein was predicted to have an Mw of 19.376 kDa and a pI of 6.26, possess a signal secretion peptide with a predicted cleavage site between amino acid residues 20 and 21 and have one predicted transmembrane helix, suggesting that SmDSR33 was a secreted protein (**Figure 1A**, **Supplementary Figure S1**).

To clarify the evolutionary relationships of this gene in different insect species, sequences of *SmDSR33* counterparts in pea aphid (*A. pisum*), soybean aphid (*Aphis glycines*), cotton aphid (*A. gossypii*), Russian wheat aphid (*Diuraphis noxia*), sugarcane aphid (*Melanaphis sacchari*), black cherry aphid (*M. cerasi*), peach aphid (*M. persicae*), banana aphid (*Pentalonia nigronervosa*), corn aphid (*Rhopalosiphum maidis*), bird cherry-oat aphid (*Rhopalosiphum padi*), and yellow sugarcane aphid (*Sipha flava*) were obtained by BLAST against aphidbase database and NCBI. The phylogenetic tree of *SmDSR33* was constructed *via* MEGA X software. Phylogenetic analysis demonstrated *SmDSR33* was more closely related to its orthologs in the pea aphid (*A. pisum*) (**Figure 1B**).

We the used qRT-PCR to investigate the *SmDSR33* expression level in grain aphids at different developmental stages. Results revealed that *SmDSR33* transcription was accumulated throughout the developmental phases at different levels (**Figure 1C**). The *SmDSR33* expression pattern peaked in the adult aphid and was about 1.6-fold higher compared to first instar nymphs.

### Wheat plants expressing *SmDSR33*-dsRNA induce *SmDSR33* silencing in aphids upon feeding

**Figure 2:**
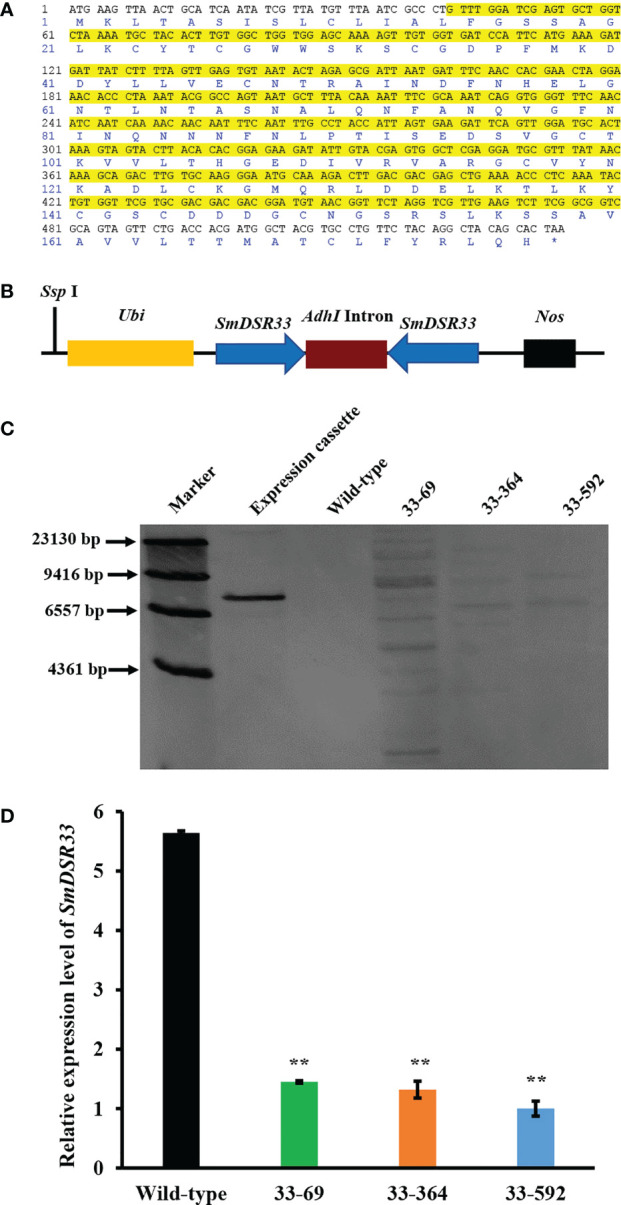
RNAi induced silencing of *SmDSR33* gene in wheat. **(A)** The encoding sequence of SmDSR33 and its deduced amino acid sequence. The sequences selected for construction of the RNAi vector are highlighted in yellow. **(B)** A schematic show of the *SmDSR33* expression cassette and position of *Ssp I* restriction enzyme. **(C)** Southern blot analysis of the transgenic wheat lines. Genomic DNA was digested with *Ssp*I and hybridized with a *SmDSR33* gene fragment with the expression cassette digested with *Ssp* I as a positive control. **(D)** Relative expression levels of *SmDSR33* of grain aphid fed on wild-type and transgenic wheat lines. The expression level of *SmDSR33* in the adult aphids fed on wild-type and different transgenic wheat lines after inoculation of one-day-old newborn nymphs, respectively. Values and error bars represent the mean and SEM of three independent biological replicates, each with a pool of 15 surviving individual aphids (Student’s *t*-test, ** *P*<0.01).

To investigate the function of *SmDSR33*, a 439 bp fragment of *SmDSR33* gene was selected as a template for RNAi target (**Figure 2A**). We used BLAST against the NCBI database to evaluate the specificity of the *SmDSR33* fragment. At the nucleotide acid level, no continuous three 21-nt matches were detected between the selected 439 bp fragment and aphid natural enemies or humans (data not shown), which implied that the selected *dsSmDSR33* fragment would not pose potential risks to non-target organisms (Bachman et al., 2013). Then, the RNAi vector harboring *SmDSR33*-hairpin DNA was constructed (**Figure 2B**). After transformed into wheat immature embryos of wheat variety cv ZM 7698, we obtained 8 independent transgenic wheat lines, among which, we randomly selected 3 of them for further analysis. Southern blot analysis indicated that the expression cassette of *SmDSR33*-dsRNA had been successfully integrated into the wheat genome with two to twelve copies (**Figure 2C**).

To further investigate whether the expression of the target *SmDSR33* gene in aphids was inhibited when feeding on transgenic wheat plants. The individual synchronous one-day-old nymphs were transferred to wild-type and transgenic wheat plants, respectively. The relative expression levels of *SmDSR33* were detected in adult aphids. The relative expression levels of *SmDSR33* in grain aphids decreased significantly upon feeding on three transgenic wheat lines (*P*< 0.01, **Figure 2D**).

### Fitness of the aphids fed on *SmDSR33-*dsRNA expressing transgenic wheat lines

**Figure 3:**
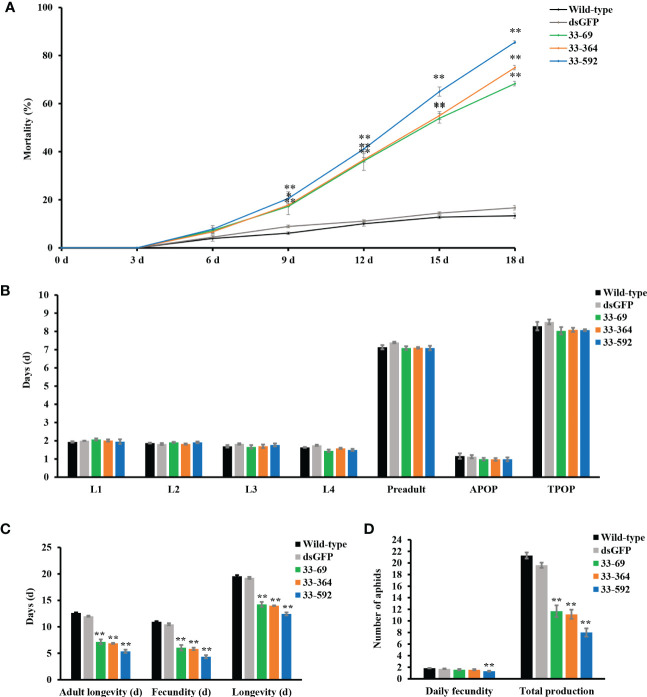
Fitness analysis of aphids fed on transgenic plants. **(A)** Mortality of aphids fed on wild-type and transgenic wheat lines. The mortality of aphids fed on wild-type and *dsSmDSR33* expression transgenic wheat lines. Twenty synchronous one-day-old nymphs were put into clip cages individually on transgenic and wild-type wheat plants. All experiments were repeated three times. Values and bars represent the mean ± SEM (Student’s *t*-test, * *P*<0.05, ** *P*<0.01). **(B)** The longevity of different stages, adult preoviposition period (APOP) and total preoviposition period (TPOP) of aphids fed on transgenic lines and wild-type control. **(C)** The adult longevity, fecundity and the total longevity of aphids fed on transgenic wheat lines and wild-type control. **(D)** The reproduction of aphids fed on transgenic wheat lines and the wild-type control. All experiments were repeated three times, each with 20 synchronous one-day-old nymphs. Values and bars represent the mean ± SEM (Student’s *t*-test, * *P*<0.05, ** *P*<0.01).

Fitness parameters including life cycle and mortality of aphids upon feeding on different transgenic lines were further investigated to evaluate the silencing impact of *SmDSR33*. The mortality rates of aphids fed on transgenic wheat lines significantly increased when compared to that of aphis fed on wild-type plants at 9 days after feeding (DAF), reaching more than 60% at 18 DAF (**Figure 3A**). We also monitored the development duration of aphids from the nymphal to imago stage. The adult preoviposition period (APOP) and the total preoviposition period (TPOP) of aphids showed no significant difference between host plant lines (**Figure 3B**). The aphid longevity fed on transgenic wheat lines was significantly shorter than on wild-type plants. Similarly, the adult longevity and reproductive period of aphids significantly decreased than wild-type (*P*<0.01) (**Figure 3C**). Consequently, in comparison with the wild-type plants, the aphid total production significantly decreased when fed on all three transgenic wheat lines (*P <*0.01) (**Figure 3D**), and the daily fecundity of aphids fed on 33-592 transgenic line decreased at a significant level (*P <*0.01) (**Figure 3D**).

All of the population parameters, including the net reproductive rate (R_0_), mean generation time (T), the intrinsic rates of increase (r_m_) and doubling times of the population (DT), showed differences between grain aphids fed on transgenic lines and those on wild-type plants (**Table 1**). For example, the net reproductive rates (R_0_) of aphids were significantly lower when fed on transgenic wheat lines. The mean generation time (T) of aphids fed on 33-592 line was significantly decreased than wild-type (*P <*0.01) (**Table 1**).

**Table 1:** Life table parameters of grain aphids fed on wild-type and different transgenic wheat lines.

Parameters	Wild-type	33-69	33-364	33-592
R_0_	21.27 ± 0.52	11.32 ± 1.08^**^	10.86 ± 0.73^**^	7.94 ± 0.77^**^
T	14.49 ± 0.30	13.40 ± 0.19^*^	13.38 ± 0.16^*^	12.57 ± 0.26^**^
r_m_	0.21 ± 0.01	0.18 ± 0.01^*^	0.18 ± 0.01^*^	0.16 ± 0.01^*^
λ	1.24 ± 0.01	1.20 ± 0.01^*^	1.20 ± 0.01^*^	1.18 ± 0.01^*^
DT	3.29 ± 0.08	3.86 ± 0.16^*^	3.91 ± 0.15^*^	4.25 ± 0.23^*^

All data are expressed as means ± SEM based on 3 repeated experiments. R_0_, net reproductive rate; r_m_, the intrinsic rate of increase; λ, the finite rate of increase; T, the mean generation time; DT, Doubling time (day). Student’s t-test, n=3, ^*^P<0.05, ^**^P<0.01.

### Feeding behavior of aphids feeding on transgenic wheat plants

**Figure 4:**
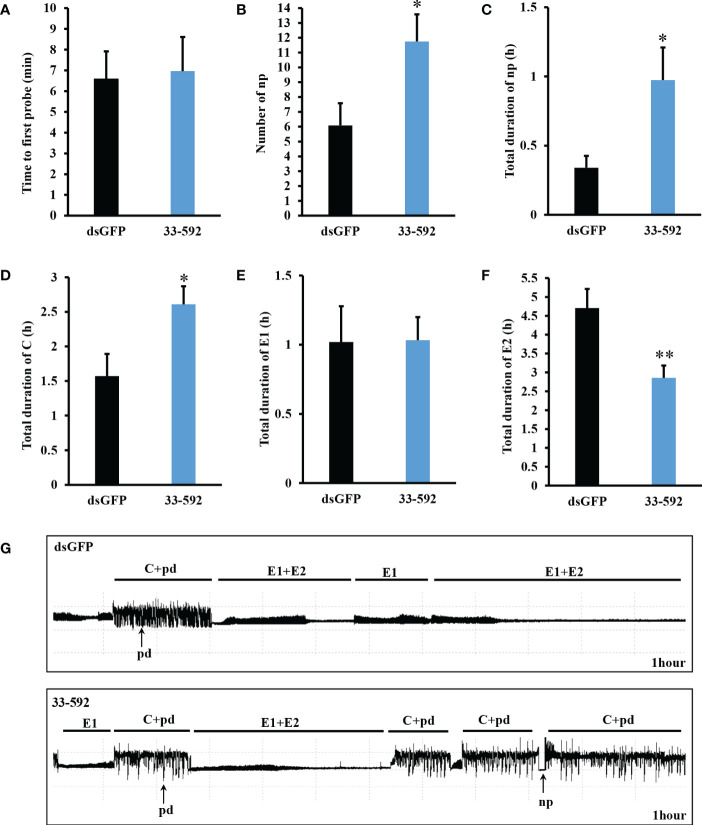
Effects of *SmDSR33* silencing on feeding behavior of grain aphid based on EPG recordings. **(A–F)** Representative parameters of aphid feeding behavior. Non-probing (np), stylet probing **(C)**, intracellular stylet puncture (pd), phloem salivation (E1), and phloem ingestion (E2). Data shown are mean ± SEM. Asterisks above bars indicate significant differences between controls and treatments (Student’s *t*-test, * *p* < 0.05; ** *p* < 0.01). **(G)** Representative EPG waveforms of grain aphids feeding on dsGFP wheat plants and 33-592 transgenic wheat plants.

To investigate the feeding behavior of aphids, transgenic wheat line 33-592 was selected to perform electropenetrography (EPG) assays. As shown in **Figures 4A-F**, there was no difference between the aphids fed on *SmDSR33* and *dsGFP* wheat plants at time point of first probe activity. The number of non-probing (np) waveforms of *SmDSR33*-silenced aphids fed on 33-592 line was significantly higher than on wild-type plants. Furthermore, the total duration of np waveforms and C phases of *SmDSR33*-silenced aphids was significantly increased compared to control. Finally, there was no difference in the duration of E1 waveforms, but did of phloem ingestion (E2) with a significant reduction for aphids on control plants. These results indicated that the feeding behavior of grain aphids was affected after *SmDSR33* silencing.

### Feeding on transgenic lines induces transgenerational silencing of *SmDSR33* in aphids

**Figure 5:**
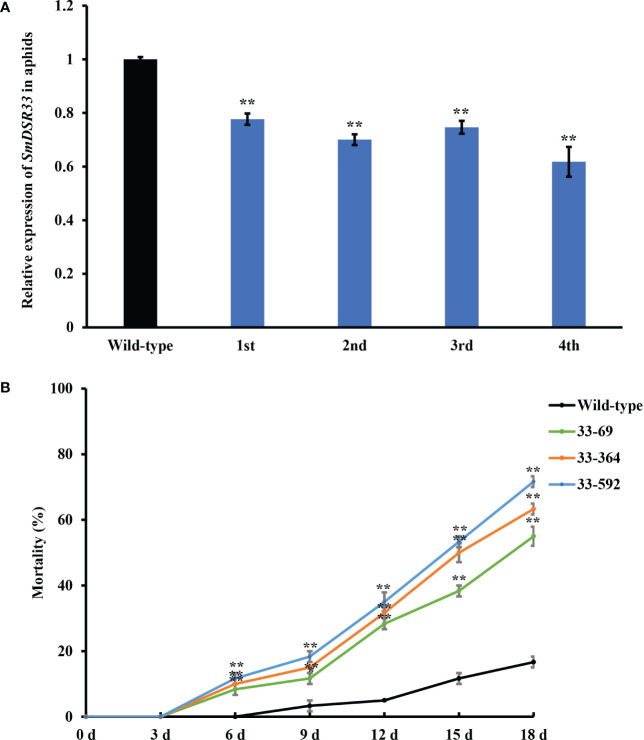
Transgenerational effect of *SmDSR33* silencing. The one-day-old newborn nymphs of aphids feeding on transgenic wheat lines were transferred to fresh wild-type wheat plants and subsequently allowed to reproduce on wild-type. **(A)** The *SmDSR33* transcript levels of adult aphids were determined in fourth successive aphid generations. **(B)** The mortality of the first generation of the offspring of aphids fed on transgenic lines at different time points after being switched to wild-type plants. Values and bars represent the mean ± SEM (Student’s *t*-test, * *P*<0.05, ** *P*<0.01).

Newborn nymphs produced in a parallel experiment were used to investigate potential transgenerational RNAi effects of *SmDSR33*. The expression levels of *SmDSR33* in the offspring of aphids fed on transgenic and wild-type plants was investigated subsequently. *SmDSR33* expression in grain aphids was suppressed in their offspring fed on wild-type plants (**Figure 5**). Aphid relative expression levels reached 77.71%, 70.04%, 74.63%, and 61.80% of control level in successive first to fourth generations (**Figure 5A**). Even after switching to wild-type plants, aphid offspring still exhibited higher mortality rates (**Figure 5B**).

## Discussion

Aphids are phloem-feeding insects that secrete saliva effectors into plant cells to enable successful feeding (Wang et al., 2015b). Salivary proteins play important roles in the interaction of aphids with plants (Yang et al., 2018; Zhang et al., 2021). Engineering transgenic plants expressing dsRNA for insect pest management is an effective strategy in agricultural practice (Ghag, 2017). Plant-mediated RNAi has been recognized as one of the most promising technologies to engineer insect-resistant crops, especially for wheat aphid control, which has great significance for food security, human health, and the agroecosystem in a global context (Sun et al., 2019; He, 2022).

Here, we identified a novel potential RNAi target gene (*SmDSR33*) from grain aphid, which had a high mortality due to the silencing of *SmDSR33* in grain aphid *via* artificial diet feeding assays (Wang et al., 2015a). *SmDSR33* was predicted as a gene encoding a secreted putative salivary protein which had a signal peptide and one predicted transmembrane helix (**Figure 1A**, **Figure S1**). This result is in accordance with previous studies on salivary effectors. For example, ApC002 was predicted to be a signal peptide for an extracellular protein and the cleavage site was predicted between residues 23 and 24 (Mutti et al., 2008). A signal secretion peptide with cleavage sites either between Ala20 and Gln21 (SignalP) or between Ser22 and Arg23 (PSORT) was predicted in Armet (Wang et al., 2015b). A secretory signal peptide at the N-terminal of the protein ACYPI006346 was predicted, with the predicted cleavage site between residues 19 and 20 (Pan et al., 2015). The signal peptide of Sm9723 was constituted of the first 21 amino acids and the cleavage site was predicted between residues 21 and 22 (Zhang et al., 2022a). The signal peptide of Sg2204 was constituted of the first 25 amino acids and the cleavage site was predicted between residues 25 and 26 (Zhang et al., 2022b).

We then obtained stable transgenic wheat lines expressing dsRNA of *SmDSR33* in grain aphids. Significantly decreased fecundity, survival, and reproduction rates of aphids fed on transgenic wheat plants were observed than that of wild-type plants (**Figure 3**). Our results are in consistent with the plant-mediated RNAi experiments targeting salivary protein and effector encoding genes in aphids. For example, silencing the salivary protein gene *C002* reduced the reproduction and survival in the pea aphid (Mutti et al., 2006; Mutti et al., 2008). Silencing salivary proteins such as Mp10, Mp42, Mp56, Mp57, and Mp58 in tobacco caused reduced virulence and fecundity of green peach aphids (Bos et al., 2010; Elzinga et al., 2014; Rodriguez et al., 2014). Silencing *Sm9723* and *Sg2204* through a nanocarrier-mediated dsRNA delivery system negatively impacted aphid survival rates and fecundity of aphids (Zhang et al., 2022a; Zhang et al., 2022b).

We found that *SmDSR33* silencing increased the total duration of non-probing waveforms and C phases and decreased the duration of phloem ingestion (E2) (**Figure 4**). These results indicated that *SmDSR33* affected the feeding process and behavior of grain aphids. It was shown that interference of target genes could affect the feeding behavior of aphids. Knockdown of an effector protein Armet impeded the feeding behavior of pea aphids (Wang et al., 2015b). As an important multipeptide molecule, neuropeptide F (NPF) had been discovered in numerous insect species and regulated a variety of physiological activities. The probing time and total duration of phloem activity on broad bean plants were decreased when wingless adult pea aphids were injected with NPF dsRNA (Li et al., 2018). When feeding on *A. thaliana*, *Mp1* silencing decreased the fitness of green peach aphids. However, aphid feeding ability with *Mp1* silences was still retained (Wang et al., 2021). Plastid-mediated RNAi was also an efficient approach for aphid control. *M. persicae* exhibited different feeding behaviors on nuclear-mediated RNAi transgenic plants and transplastomic-mediated RNAi transgenic plants (Dong et al., 2022). Feeding behavior of *S. miscanthi* and *S. graminum* were significantly impaired after knockdown of *Sm9723* and *Sg2204* (Zhang et al., 2022a; Zhang et al., 2022b).

According to previous studies on environmental RNAi, transgenerational silencing, also known as parental RNAi, in which the silencing effects of the respective target genes and survival rates could be significantly impacted in the offspring of the treated organism (Marré et al., 2016; Rechavi and Lev, 2017; Wang and Hunter, 2017). Our data showed that *SmDSR33* relative expression levels reached from 78 to 62% of control level in the following first to fourth successive generations (**Figure 5**). This result indicated that RNAi effect was persistent in grain aphids. This type of effect could last for several days, many weeks, few months, and even for multiple generations. With time and successive generations, the silencing effect decreased (Amdam et al., 2003; Jaubert-Possamai et al., 2007; Miller et al., 2012; Abdellatef et al., 2015). According to a previous study, parental RNAi may result from a specific dsRNA uptake mechanism or small amounts of incidentally incorporated dsRNA secondary amplification (Bucher et al., 2002). The phenomenon of telescoping generations existed in grain aphids, which means that the developing grandchildren are already carried by a parthenogenetic adult, may facilitate the transfer of siRNA/dsRNA to the subsequent generations in aphids (Abdellatef et al., 2015). Transgenerational silencing could also induce by small RNAs mediated epigenetic modifications (Castel and Martienssen, 2013). We observed the decreased silencing effect in 4^th^ generation compared to that of 1^st^ to 3^rd^ generation. Our result was consistent with the study that the duration of the RNAi impact was doubled in nymphs whose mothers had been exposed to dsRNA-producing transgenic plants (12-14 days), which indicated that the RNAi effect may persist longer in nymphs than in their mothers (Coleman et al., 2015). This could be due to the fact that the stability of dsRNA in insects may be affected by the quantities of dsRNA, the lengths of the dsRNA fragments, the activities that degrade dsRNA, and the life stages of the target species (Griebler et al., 2008; Huvenne and Smagghe, 2010; Bolognesi et al., 2012; Miller et al., 2012; Abdellatef et al., 2015). Transgenerational gene silencing exhibited significant potential in RNAi-mediated pest control, although the molecular mechanisms in insect species remained to be elucidated.

In conclusion, we not only identified and characterized a novel RNAi target gene *SmDSR33*, which is a putative salivary secretion protein in grain aphids, but also revealed that targeted silencing of *SmDSR33 via* plant-mediated RNAi significantly decreased the survival, fecundity, and total production of grain aphids, which consequently reduced aphid infestation on wheat plants. The altered feeding behavior and transgenerational RNAi silencing effects also minimized aphid infestation. As a result, our study demonstrated the significant potential of plant-mediated RNAi of an important putative salivary protein gene as a promising strategy for aphid control in crop plants in agricultural practice.

## Data availability statement

The raw data supporting the conclusions of this article will be made available by the authors, without undue reservation.

## Author contributions

FF and LX conceived and designed the experiments. JZ, HL, XZ, JT and AS performed the experiments. JZ analyzed the data. FF, LX and JZ wrote the manuscript. FF and LX revised the manuscript. All authors read and approved the final manuscript.
