# Supplementary material for: Silencing an aphid-specific gene SmDSR33 for aphid control through plant-mediated RNAi in wheat
Source: Front Plant Sci. 2023 Jan 9;13:1100394. doi: 10.3389/fpls.2022.1100394 (PMC9868936; doi:10.3389/fpls.2022.1100394)
Supplement: Supplementary file 1 [file DataSheet_1.docx]

Supplementary Material

**Contents**

**Supplementary Figure 1.** The predicted signal peptide and transmembrane helix of SmDSR33

**Supplementary Table 1.** The primers used in this study

**(A)**


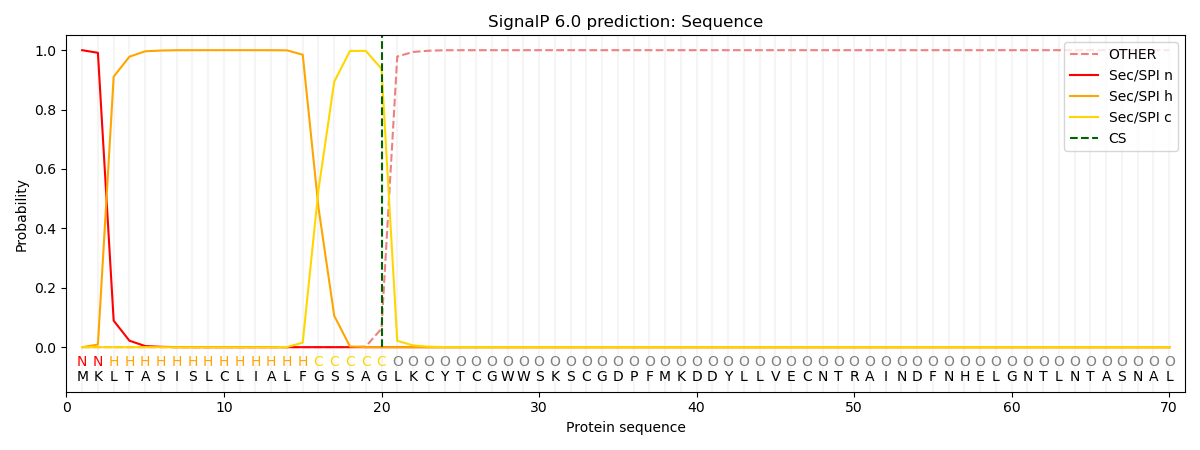


**(B)**


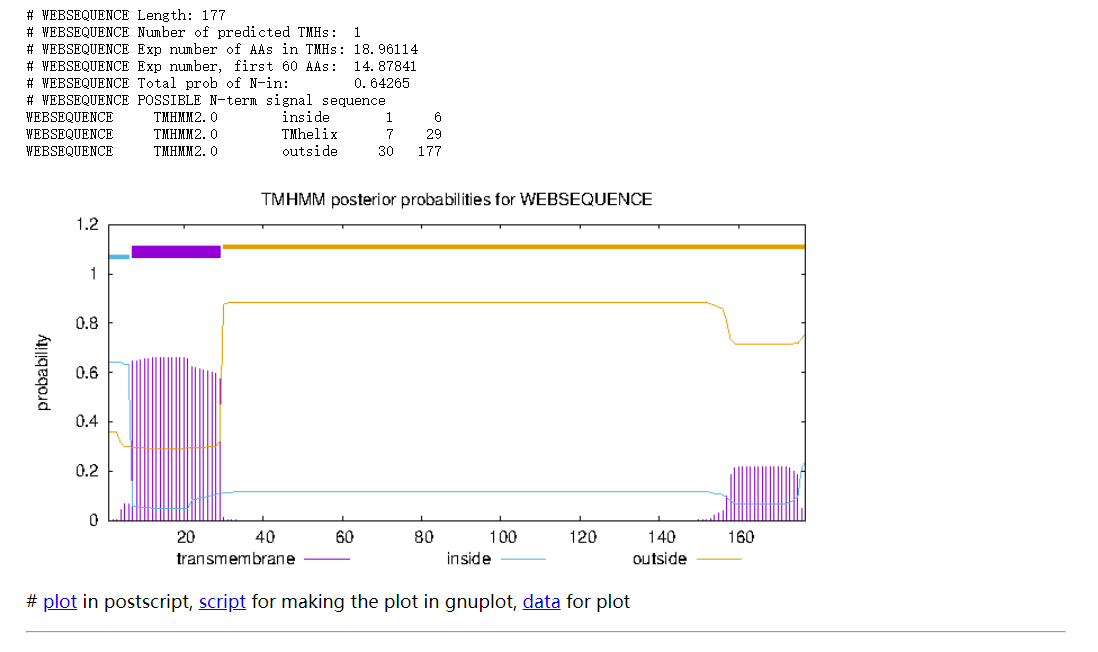


**Supplementary Figure 1. The predicted signal peptide and transmembrane helices of SmDSR33**

(A) The predicted signal peptide of SmDSR33. The cleavage site is between residues S^18^A^19^G^20^-L^21^K^22^.

(B) The predicted transmembrane helix of SmDSR33.

**Table S1. The primer sets used in this study**

| **Primer** | **Sequence (5′ to 3′)** | **Use of PCR products** | **Annealing (℃)** | **Fragment size (bp)** |
| --- | --- | --- | --- | --- |
| SmDSR33c-F | atgaagttaactgcatcaatatcgttatg | Full length cDNA cloning | 56 | 534 |
| SmDSR33c-R | ttagtgctgtagcctgtagaacag |  |  |  |
| SmDSR33S-F | ATCCCCGGGGGATCCACTAGTgtttggatcgagtgctggtct | Construct vector for RNAi | 59 | 483 |
| SmDSR33E-R | GCCTCCCAGATCGATTCGATATCgaccgccgaagacttcaacg |  |  |  |
| SmDSR33H-F | GGGGCAGACTCCCGTTTGTTAACgaccgccgaagacttcaacg | Construct vector for RNAi | 59 | 483 |
| SmDSR33S-R | CGATCGGGGAAATTCGAGCTCgtttggatcgagtgctggtct |  |  |  |
| qactin-F | CGGTTCAAAAACCCAAACCAG | Internal control of qRT-PCR for aphid | 56 | 260 |
| qactin-R | TGGTGATGATTCCCGTGTTC |  |  |  |
| Rps27-F | TGTGAAGACGTTGACTGGGAAA | Internal control of qRT-PCR for aphid | 57 | 114 |
| Rps27-R | CGTTGCTGATCCGGAGGAATAC |  |  |  |
| SmDSR33S-F | CGACGAGTCTAACGGACACC | Southern blot | 58 | 456 |
| SmDSR33S-R | CCCTAACCATGGACCGGAAC |  |  |  |
| SmDSR33q-F | CCCTGTTTGGATCGAGTGCT | Detect *SmDSR33* in aphids | 57 | 150 |
| SmDSR33q-R | GGGTGTTTCCTAGTTCGTGGT |  |  |  |
| Ubi-F | GCCTTCATACGCTATTTATTTGCTTGGTAC | Detect *SmDSR33* dsRNA in transgenic wheat | 60 | 687 |
| ADH1-R | GGAGTCTGCCCCTAAGACAGATAAGC |  |  |  |
| ADH1-F | CTGGGAGGCCAAGGTATCTAATCAGC | Detect *SmDSR33* dsRNA in transgenic wheat | 60 | 647 |
| Nos-R | CGCAAGACCGGCAACAGGATTC |  |  |  |
